# Co‐Design of a Framework for Person‐Centred Care at Emergency Department Triage and Waiting Room

**DOI:** 10.1111/hex.70442

**Published:** 2025-09-22

**Authors:** Carrie Janerka, Gavin D. Leslie, Fenella J. Gill, PCC ED Triage Group, Louise Brincat, Amber Cooper, Leeuwin Glosz, Libby McNeilly, Charlie Rhodes, Kristy Sidrak, Emma Smith, Amelia Toffoli, Louise Ward, Sharon Waterson, Jayne Woodley‐Page

**Affiliations:** ^1^ School of Nursing Curtin University Bentley Western Australia Australia; ^2^ Nursing and Midwifery Research Unit South Metropolitan Health Service Murdoch Western Australia Australia; ^3^ Perth Children's Hospital Child and Adolescent Health Service Nedlands Western Australia Australia

**Keywords:** emergency nursing, emergency service, hospital, patient‐centred care, triage, waiting rooms

## Abstract

**Introduction:**

Patients presenting to an emergency department (ED) are typically assessed by a triage nurse and often required to wait for further assessment and care. Ongoing issues of ED overcrowding and prolonged wait times can impact the processes and patients' experiences. A person‐centred approach is recommended. This study aimed to develop a person‐centred care framework for use in the ED triage and waiting room.

**Methods:**

The multi‐phase research involved a synergistic partnership‐based fully integrated mixed‐methods approach based on person‐centred care principles. Framework development followed target population‐centred and partnership‐based processes of conception, planning and designing. A consumer group was actively involved throughout. Data collection involved two literature reviews, a patient survey and triage nurse survey. Findings were synthesised through focus group sessions and the framework was developed during a collaborative workshop.

**Results:**

A total of 225 patients and 176 triage nurses responded to the respective surveys. Patients reported the need for better communication, efficiencies and comfort in the waiting room. Nurses identified barriers to person‐centred care, such as workloads, poor environment and long wait times. Strategies for overcoming barriers included enhanced communication, addition of waiting room staff and family involvement. Focus groups recommendations were synthesised as support for staff, systems and processes, environment and facilities, communication and information, and individual care needs, forming the framework elements/components. Practical micro, meso and macro level interventions were also recommended.

**Conclusions:**

The newly developed framework can now be applied to ED triage settings and inform person‐centred interventions.

## 1 Introduction

Triage is an essential first point of contact for patients who present or are brought to the emergency department (ED). It typically involves evaluation by a triage nurse to determine the patient's clinical urgency and/or resources required for their care using a standardised triage system or scale [1, 2]. Ongoing issues of ED overcrowding, access block and staffing challenges have disrupted triage processes, wait times and patient care [3]. Various initiatives, such as time targets, patient streaming, fast track pathways, nurse‐initiated interventions and team triage, have been implemented in an attempt to address and improve patient throughput, with varying success [3, 4]. However, a focus on patient flow can negatively impact patient and staff interactions and staff's ability to provide non‐technical, interpersonal aspect of care [5]. High volumes of ED presentations and prolonged wait times continue to challenge triage nurses to provide optimal and timely care, which in turn can impact patients' experiences and subsequently their likelihood to wait for medical assessment or return to the ED [6]. A person‐centred approach at triage and waiting areas has been recommended [6].

Models of care founded upon the principle of ‘centredness’ have become a prominent movement in healthcare, emphasising the importance of placing the person, rather than the illness, at the centre of care [7]. A centred approach to care can improve patients' physical and social wellbeing, quality of life and satisfaction with care, as well as reduce hospital admissions and cost of care [8, 9, 10]. Patient‐centred, person‐centred and people‐centred approaches have been adopted widely, and whilst the terms share commonalities and are sometimes used interchangeably, they have distinct philosophical emphases. Patient‐centred care involves empathy and respect, meeting individual patients' needs, communicating with and engaging them in decisions and care, and providing individualised, holistic and coordinated care in the clinical context [11]. Person‐centred care shares similar elements but is underpinned by a humanistic philosophy that positions the individual as a person, considering their values and lived experiences, and a focus on creating authentic engagement and healthful relationships between patients, their significant others and care providers [12, 13]. People‐centred care, promoted by the World Health Organization [14], takes a broader systems‐level view, addressing community and population needs alongside individual care. Whilst patient‐centred care has been a dominant concept historically, catalysed by the Institute of Medicine's 2001 report for quality healthcare [15], organisations globally have recently transitioned towards promoting a person‐centred approach [10, 16, 17, 18, 19].

This study is primarily underpinned by conceptual frameworks for person‐centred care, specifically the Picker Principles for Person‐centred Care [16], whilst acknowledging the historical influence of patient‐centred approaches [10]. The Picker principles emphasise respect for patients' values and preferences, coordination and integration of care, clear communication and information, physical comfort, emotional support, involvement of family and friends, continuity and transition and access to care. Operationalising person‐centred care is complex and requires understanding contextual and relational factors, and supporting person‐centred practice at micro, meso and macro levels [10, 12]. In the ED, a focus on medical‐technical priorities, patient flow, overcrowding, a stressful environment and limited staff training have posed barriers to the provision of person‐centred care [20, 21]. In the triage and waiting room context, these challenges may be compounded; patients may experience pain, anxiety and prolonged wait times, while receiving limited or unclear information, which can negatively impact their care experience [6].

The unique environment of the ED triage and waiting room setting requires a considered approach to person‐centred care, yet guidance in this context is lacking. Current person‐centred models provide broad elements, requirements and expectations for person‐centred care [16, 22, 23] but their application at ED triage remains unclear. A pragmatic approach to guiding and implementing person‐centred care in this setting is needed. Interventions should be informed and designed in partnership with those delivering and receiving the care to support their applicability and acceptability by stakeholders [24]. Person‐centred interventions should be considered and supported at micro (clinical), meso (organisation) and macro (health system) levels [10]. The aim of this study was to develop a person‐centred care framework for use in the ED triage and waiting room context. This paper describes the process and synthesis of four studies undertaken in collaboration with patient and nurse consumer groups to develop the framework.

## 2 Materials and Methods

### 2.1 Design

**Figure 1:**
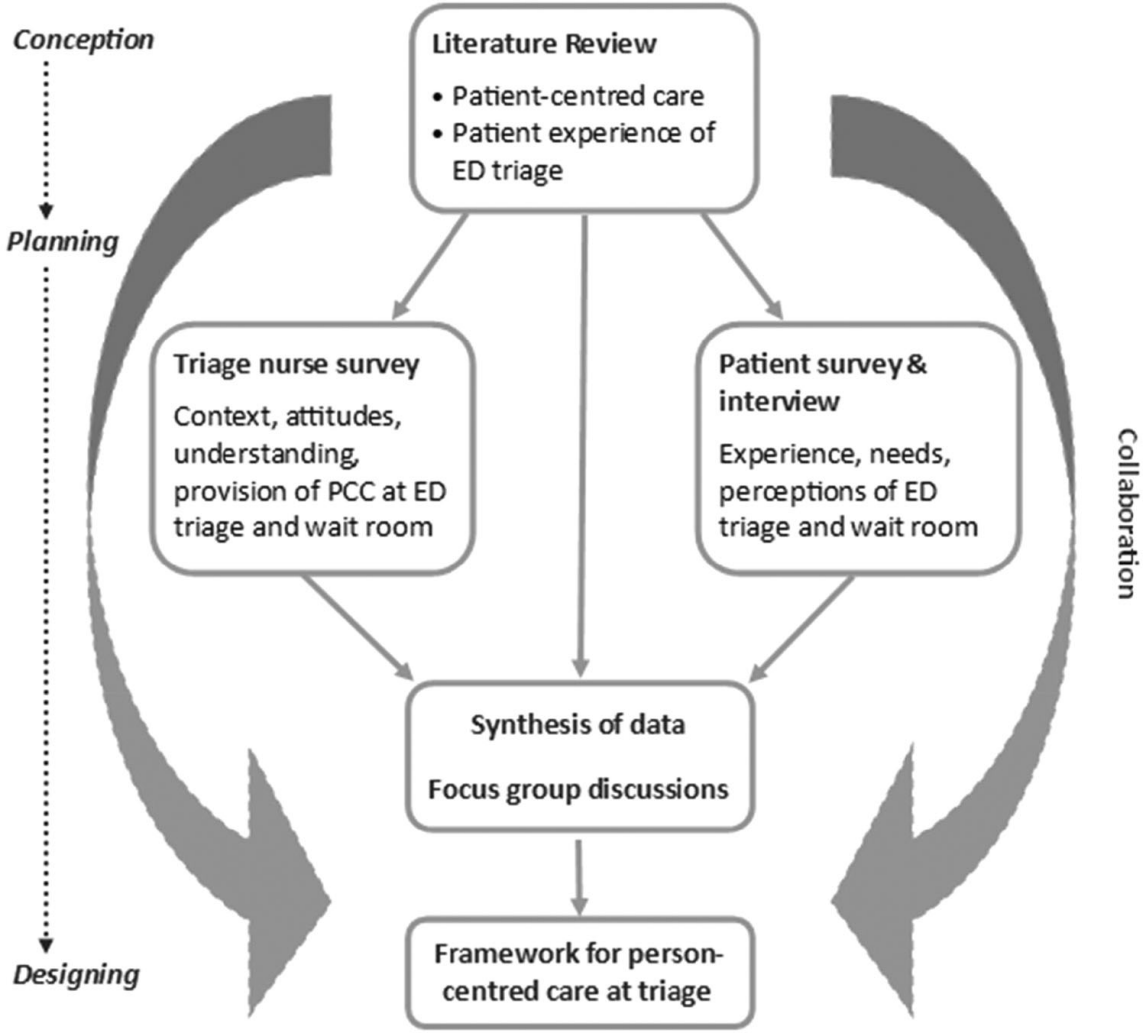
Research process.

Driven by a pragmatic worldview that considers real‐world situations and solutions and tailoring research methods to suit the question, the research involved multiple methods [25]. Nastasi and colleagues' synergistic partnership‐based fully integrated mixed methods framework [26] guided the research design and supported the collection and synthesis of data from various perspectives, across multiple phases, in collaboration with a consumer group (Figure 1). Development of the person‐centred framework followed O'Cathain and colleagues' seven‐stage process for health intervention development and utilised a target population‐centred and partnership approach [24]. The first three stages involve comprehensive concept development, whilst the final four stages guide operational testing. This study involved the first three stages of conception, planning and designing the framework. The framework was co‐designed with patients and nurses (in partnership) and is based on the views and actions of users and receivers of the intervention (target population‐centred) [24]. Reflexivity was maintained by the lead researcher (C.J.) throughout the project by regularly reflecting on the research processes and findings with the research supervisors (F.J.G. and G.D.L.) and the consumer group. Reporting of the research follows the Good Reporting of A Mixed Methods Study (GRAMMS) guidelines [27].

### 2.2 Consumer Involvement

Collaboration with consumers was integral to the success of the research. A consumer reference group of five patients and five triage nurse representatives was established and actively involved throughout the 5‐year research [28]. The consumer reference group collaborated in decision‐making for key aspects including ethical considerations, survey development and testing, participant recruitment, interpretation of research findings and design and creation of the framework. Group members lived experience, unique perspectives and understanding of real‐world practices and processes helped to ensure research quality and relevance and support future knowledge translation [29].

### 2.3 Stage 1: Conception

The first stage involved identification of the problem and need for a solution. A concern that some patients had suboptimal experiences of presenting to the ED and waiting in the waiting room was identified through experience of the author, colleagues, patient anecdotes and by a local ED consumer advisory group. It was noticed that care at triage and in the waiting room was less person‐centred than care provided inside the main department. Synthesis of published literature validated the concern and helped to explore the problem and identify relevant concepts. A meta‐narrative review of 124 articles published between 2012 and 2021 related to patient‐centred care in acute care settings, and an integrative review of 29 articles published between 2000 and 2022 reporting patient experience of ED triage and/or waiting room were conducted [6, 10]. Results of the two literature reviews informed survey tool development for the next stage.

### 2.4 Stage 2: Planning

The ‘planning’ stage comprised the establishment of a group to guide the development process and understanding the problem or issues to be addressed through data collection and investigation. A consumer reference group was formed and was involved throughout. In addition, stakeholders at each site were engaged during data collection; they included nurse managers, department heads, site investigators and a local ED consumer advisory group. Data collection involved two cross‐sectional surveys and an interview. Methods and results for both surveys are presented in summary in this paper, with detailed reporting elsewhere [30].

#### 2.4.1 Patient Survey

A patient survey was conducted across five Australian EDs to understand patients' experiences of presenting to an ED, interacting with the triage nurse and waiting for a medical assessment in the waiting room. The purpose‐designed survey was tested for clarity, content validity, apparent internal reliability, included quantitative data items exploring person‐centred elements across their journey, such as level of anxiety on presentation, whether the triage nurse listened, and if they were provided pain relief, to identify what needs to change, as well as qualitative suggestions for change. Quantitative data were analysed descriptively and explored for correlations. A conventional content analysis approach was used to analyse qualitative data.

#### 2.4.2 Patient Interview

Researchers planned to conduct narrative interviews with patients who responded to the survey and agreed to an interview, to better understand survey results and the patient journey. One interview was conducted. However, challenges of patients being unwell, unavailable or not wanting to re‐live their experience were encountered early on and so it was agreed, in partnership with the consumer reference group, not to recruit further interview participants.

#### 2.4.3 Triage Nurse Survey

A triage nurse survey was distributed at the same five ED sites, as well as nationally via the College of Emergency Nurses Australasia. The purposefully developed survey was tested for clarity, content validity apparent internal reliability and collected data about the ED and triage features as well as nurses' preparation, knowledge, attitudes and provision of person‐centred care at triage. The purpose of the survey was to understand triage nurses' perspectives on the existing culture, contexts and supports for person‐centred care at triage, including barriers and strategies for its provision. Quantitative data were analysed using descriptive statistics. Responses to open‐ended questions were analysed inductively and deductively.

#### 2.4.4 Focus Groups

Focus group sessions were conducted to reach consensus on specific problems to be addressed and identify real‐world recommendations for improvement [24]. Design and running of the focus groups followed Gray's approach of (1) identify problem, (2) recruit participants, (3) identify moderator and others, (4) book and prepare facilities, (5) generate questions, (6) conduct the group, (7) record data and (8) analyse and interpret data [31]. An invitation to participate in a 3‐h session was sent to triage nurses and consumer groups at the five ED sites, as well as snowball recruitment through the consumer reference group. Prior to their session, participants received an information booklet with venue information, an agenda, expectations of the session and a copy of the surveys. One researcher facilitated the sessions (C. J.) and a senior researcher supervised and took notes (G. D. L.). The researcher, who was an emergency nurse, ensured trustworthiness by critically reflecting on their own preconceptions (reflexivity), listening to participant's voices, and maintaining neutrality throughout data collection and analysis [32]. Results of the surveys were presented to the focus groups using a side‐by‐side comparison approach for data integration [25]. Participants were asked whether the results ‘made sense, or seemed right’, if they ‘agreed or disagreed with the results’ and to share their perspectives. After key issues were synthesised, participants were invited to suggest individual (micro), organisation (meso) and health system (macro) level recommendations for change/improvement [10] displayed as notes on a digital whiteboard.

#### 2.4.5 Data Synthesis

In keeping with the integrated synergistic mixed‐methods approach [26], inferences drawn from literature findings and quantitative and qualitative survey results were synthesised using a convergent parallel design described by Creswell and Creswell [25]. The researcher, who was immersed in the data, compared all qualitative, quantitative and literature review results to identify common ideas and synergies. Consensus recommendations from the focus group sessions were merged with relevant findings from the surveys and literature reviews using a table, then grouped into categories, with consideration of Pickers Principles of Person‐centred Care [16] and within the micro, meso and macro levels.

### 2.5 Stage 3: Designing

The third stage involved generating ideas about solutions, designing the framework and making decisions about its format and delivery. Members of the research team, consumer reference group and site investigators attended a 2‐h framework development workshop. In the first half of the workshop, attendees were presented with and discussed all research processes, key findings from the literature reviews and surveys (which they were familiar with), and focus group recommendations (new information) to ensure a complete understanding of the findings. In the second half of the workshop, the researcher proposed an initial framework prototype, modelled on similar research [33], was discussed and iteratively adapted following O'Cathain et al. [24] partnership and target population‐centred approach to intervention development ‘designing stage’. Attendees were encouraged to use their expertise to consider a range of solution and how they can work in the real world, including acceptability, usability, satisfaction and psychosocial context of the user, as well as identify objectives and features required to implement the framework and consider the priority of interventions.

### 2.6 Ethical Considerations

The research was approved by the South Metropolitan Health Service Human Research Ethics Committee (RGS0000006844) and reciprocal approval from Curtin University HREC (HRE2024‐0326). All participants were provided an information sheet and consent form, including parents or guardians where relevant. Survey data were anonymous, and interview and focus group data were de‐identified. All study documents and data were stored safely and securely.

## 3 Results

### 3.1 Patient Survey and Interview

**Figure 2:**
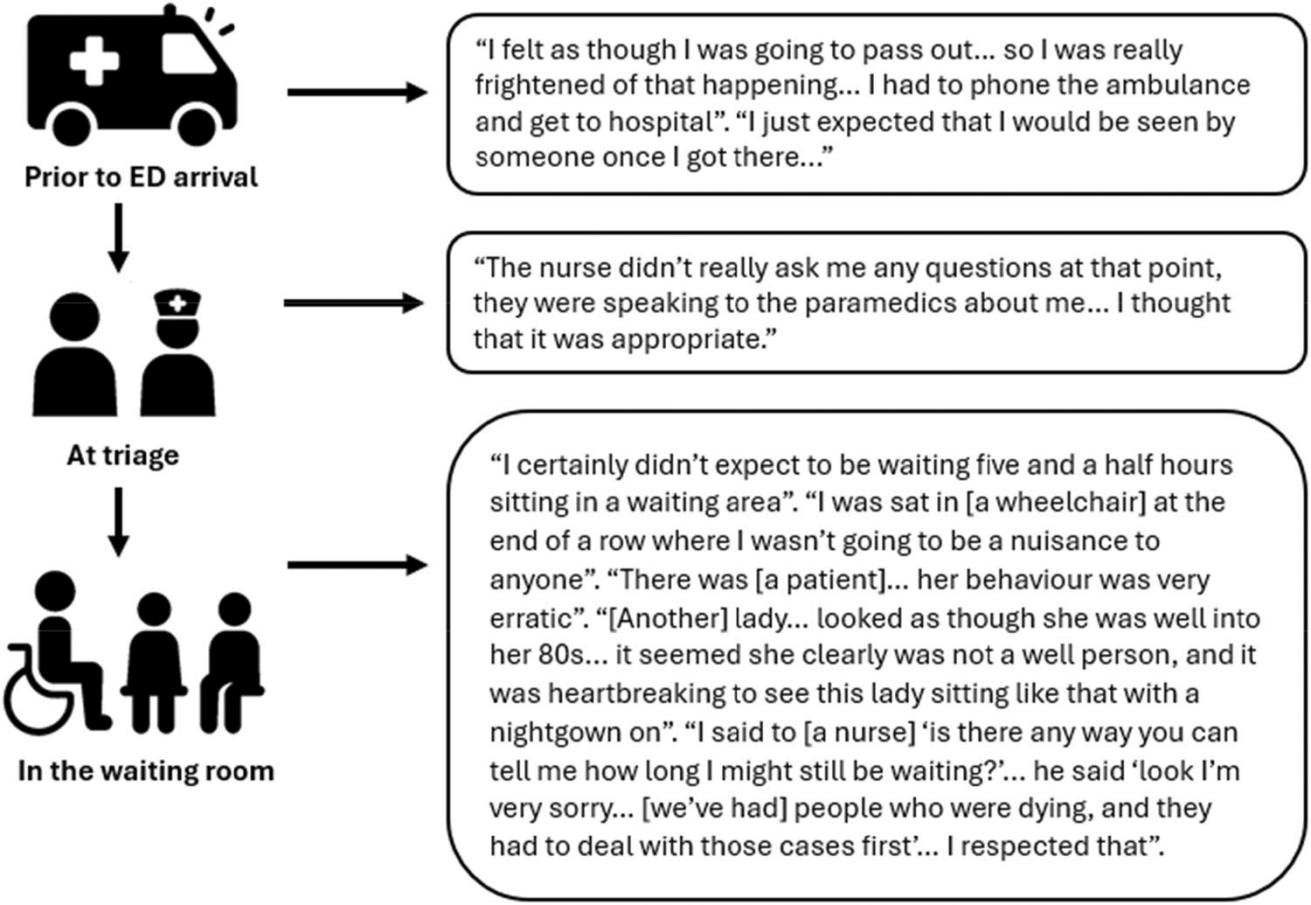
Triage and waiting room journey of Helen*, an older adult female patient in a metropolitan ED. *Helen is a pseudonym.

A total of 225 patients aged 14 years and older responded to the survey. Patients reported that on arrival, they experienced moderate–high levels of pain and anxiety (median 7/10). Satisfaction with the triage nurse was very high in most cases (median 9/10). After triage, patients reported wait times ranging from 0 to 660 min (median 60 min). Patient satisfaction was negatively correlated with wait time (*p* < 0.001). Areas for change/improvement included the provision of wait time information and pain relief. Patients suggested increased staff, more efficient processes, comfortable seating, more pleasant environment, better communication with staff and consideration of individual care needs could improve their experience. Excerpts from the patient interview provide a narrative perspective of the patient journey (Figure 2).

### 3.2 Triage Nurse Survey

A total of 176 triage nurses who worked at various sized EDs across Australia responded to the survey. A range of existing triage processes and waiting room features were reported, including patient streaming (patients are allocated to a stream based on their needs), ticket systems, nurse‐initiated interventions, ED process information provision and waiting room nurse roles. Responses showed that triage nurses largely understood concepts and valued person‐centred care, however, their provision of person‐centred care varied. Several barriers were identified and included time constraints and heavy workloads due to high volumes of patients, inadequate staffing and prioritisation of other clinical care, as well as lack of private areas, noisy environment, long wait times and their experiences dealing with frustration and aggression from patients and families. Strategies for providing person‐centred care included advanced communication and interpersonal skills, provision of ED process and wait time information to patients, enabling more timely pain management, introduction of staff roles in the waiting room and involving families in patient care activities.

### 3.3 Focus Groups

A total of nine participants (five patient representatives, four nurses) attended three focus group sessions to discuss survey results, identify areas for change/improvement and suggest pragmatic recommendations to address these. In total, 43 recommendations were made at micro, meso and macro levels and a further 10 recommendations identified from the literature reviews were added. The 53 recommendations were grouped into categories of (1) support for staff, (2) systems and processes, (3) environment and facilities, (4) communication and information and (5) individual care needs (Table 1).

**Table 1:** Pragmatic recommendations to improve patient experience of ED triage and waiting room.

Level	Category	Recommendations (interventions/strategies)
Individual (micro)	Support for staff	▪ Recognise burnout in self and in colleagues (take a break, consider wellbeing) ▪ Experienced nurses to role model/mentor new nurses (e.g., managing situations) ▪ Be aware of own emotions (can impact on communication and interactions)
	Communication and information	▪ Courteous triage communication (purposefully introduce self, ‘how can we help you?’) ▪ Use a structured guide/script at triage (e.g., ‘introduce, acknowledge, empathise, explain’) ▪ Identify the patient's concerns, provide health information and reassurance where possible ▪ Offer information about triage/ED process (i.e., sickest patients will be seen first, streams) ▪ Empower patients and family and give permission to ask for care (e.g., pain relief) ▪ Check‐in with patients regularly (provide wait time updates, explain what is happening and why, reassure ‘we haven't forgotten you’, reassess needs)
	Individual care needs	▪ Consider how you/your family would like to be treated ▪ Humanise the patient (e.g., use their name) ▪ Ask about level of anxiety and ‘what most concerns you?’ (identify expectations) ▪ Focus on individualised care for patients in the waiting room ▪ Consider the patient's past ED experience (none, traumatic) to inform care ▪ Consider ‘what can I do to improve the patient's experience’ (e.g., comfort measures) ▪ Look for patients/visitors trying to get your attention ▪ Provide timely pain relief ▪ Consider patient/visitors impressions of staff (be mindful of own behaviour)
Organisation (meso)	Support for staff	▪ Rotate staff allocations to triage roles ▪ Allocate experienced nurses with new triage/waiting room nurses for role modelling/mentoring ▪ Facilitate access to staff wellbeing services/resources (counselling, psychological first aid) ▪ Triage/waiting room training to include person‐centred care, that is, effective and empathic communication, patient perspective (e.g., patient stories, sit in the waiting room as a ‘patient’) ▪ Triage/waiting room policies and guidelines to include person‐centred care, including provision of information (e.g., triage assessment, wait time) ▪ Adequate staffing to support the provision of person‐centred care ▪ Leadership role modelling and support for person‐centred care
	Systems and processes	▪ Staff roles to initiate interventions for waiting room patients, including administering pain relief ▪ Dedicated room for starting care interventions in privacy ▪ Processes to limit the number of non‐patients in the waiting room when crowded ▪ Processes for managing disruptive behaviours by patients/visitors in the waiting room ▪ Aboriginal Health Worker to liaise with Aboriginal patients in the waiting room ▪ Ticket system for patients awaiting triage to know where they are in the queue ▪ Concierge to greet patients/visitors arriving at ED ▪ Always have someone (staff, volunteer) in the waiting room ▪ Delineated areas in the waiting room (e.g., area for fast‐track patients, children) ▪ Establish an ED Consumer Advisory Group to help guide patient experience interventions ▪ Processes to reduce time‐to‐triage during surges (additional triage nurse)
	Environment and facilities	▪ TV for entertainment (quiet, with subtitles, child‐friendly) ▪ Vending machine for food ▪ Comfortable chairs ▪ Space for patients who need to lie down ▪ Create a calming environment in the waiting room (colours, smells, white/brown noise) ▪ Make available blankets for patients in the waiting room
	Communication and information	▪ Guide/script for providing ED process and wait time information ▪ Communicate information about the ED in various forms (e.g., TV, videos, posters, website, staff), including what to expect, what to bring ▪ Provide wait time information (displayed and staff communication)
	Individual care needs	▪ Allocate workload to enable sufficient time for person‐centred care for patients in the waiting room
Health system (macro)	Support for staff	▪ Triage/waiting room training to include person‐centred care, that is, communication, patient perspective (e.g., patient stories, sit in the waiting room as a ‘patient’) ▪ Triage/waiting room policies and guidelines to include person‐centred care
	Systems and processes	▪ Staffing standards for triage (e.g., staff to patient ratios, skills and qualifications required) ▪ State/national standards support consistent triage/waiting room models and processes ▪ Involve consumers in triage policies and design ▪ Standards for time‐to‐triage and maximum wait time ▪ Establish pathways for diverting/referring patients to urgent care or GP
	Environment and facilities	▪ Standards for waiting room facilities (comfortable chairs, food, television)
	Communication and information	▪ Standard way of providing information about wait times and patient numbers at EDs ▪ Education for GPs about ED services, how and when to refer

Abbreviations: ED = emergency department, GP = general practitioner/primary care physician, TV = television.

### 3.4 Framework Design

**Figure 3:**
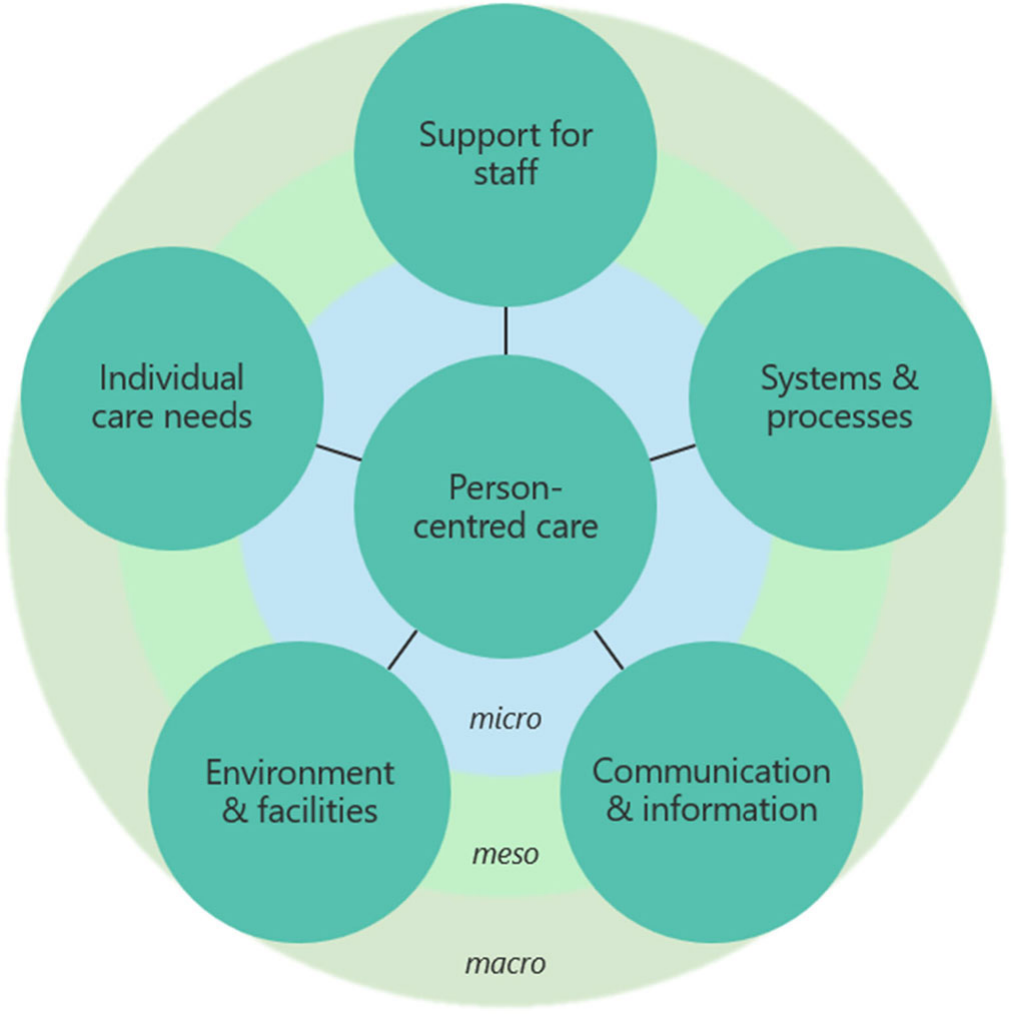
Framework for person‐centred care at ED triage and waiting room.

The workshop was attended by researchers, consumer group members and site investigators (*n* = 10). Four key ideas were agreed and provided the pragmatic basis for framework design: (1) lack of standardisation of ED triage and waiting room processes and features, (2) the need to include patients in the waiting room in nursing ratios staffing formulas, (3) the opportunity to make ‘quick fixes’ that can improve care: better chairs, provision of wait time information and ticketing systems when patients first arrive and (4) adequate staffing to provide basic needs for patients in the waiting room. In designing the framework, participants proposed a simple model that can be applied across different ED triage contexts but emphasised the need for supporting information and resources to guide its practical application. The framework (Figure 3) depicts the five key elements of person‐centred care in the ED triage context identified in Table 1 (as categories) that should be considered at the micro, meso and macro levels. Participants agreed on the importance of creating a ‘how to’ booklet that outlines possible interventions, real‐world examples and checklists for providing person‐centred care. Other recommendations were online learning modules for staff and a smart phone application with information for patients about their ED visit and what to expect.

## 4 Discussion

For this project, we used multiple research methods and consumer involvement to co‐design a framework and practical interventions for providing person‐centred care at ED triage and waiting room. The novel approach ensured the inclusion of user perspectives to design an evidence‐based, pragmatic framework and interventions tailored for the context. The framework consists of five elements: support for staff, systems and processes, communication and information, environment and facilities and individual care needs, that broadly align with the Picker Principles of Person‐centred Care [16]. Interventions for each element can be considered at the micro (individual), meso (organisation) and macro (health system) levels. Possible interventions at each level were identified and range from ‘quick fix’ solutions to structural changes and service standardisation, with a common focus of improving patient experience in the climate of ED overcrowding and prolonged wait times.

At the micro level, triage nurses can adopt communication strategies, provide information, and tend to individual patient care needs. Similarly, staff interaction with patients, provision of information, and initiation of assessment and treatment at triage have frequently been reported to influence patients' experiences of ED [6]. In this study, we found communication and information sharing were particularly important at ED triage and waiting room. The patient survey identified reported high levels of anxiety and long wait times for patients presenting to the ED, and their desire for better communication and information to help mitigate these. Acknowledging the patient experience, triage nurses and focus groups described possible communication strategies such as scripted triage conversations, offering more information, providing reassurance and regular check‐ins with patients who are waiting. Graham et al. [33] in their qualitative meta‐synthesis of patient experience in the ED, similarly reported the use of communication and interpersonal strategies to address the needs of patients in the ED, such as active listening, humour (where appropriate) and providing clear and timely information. Regularly and frequently interacting with patients can enable nurses to provide important timely information, which can in turn reduce patient anxiety [33]. In addition, increasing interactions with patients provides opportunity for nurses to understand the patient as an individual, determine their unique needs and provide consistent care.

Person‐ or patient‐centred care interventions have typically been reported as being implemented at the micro level, putting the onus on staff to adopt a new or modified approach to care [10]. Many national and international models encourage health professionals to involve and empower patients, provide holistic care and treat patients with dignity and respect [14, 18, 34]. Some models overlook the physical and emotional wellness of staff who are essential for consistently maintaining this standard of care, particularly in high turnover, customer‐facing environments such ED triage. McCance and McCormack [12] emphasise the need to consider nursing attributes as a prerequisite for providing effective person‐centred care. Our research clearly identified the need for staff to consider their own wellbeing, as well as that of their colleagues, to support their provision of quality patient care. This involved being aware of emotions and burnout in self and others and utilising experienced nurses to role model person‐centred behaviours. Burnout has been recognised amongst ED nurses, linked with busy work schedules, violence towards staff, lack of management support and poor work environments [35, 36]. Triage nurses in our research similarly reported heavy workloads, inadequate staffing and aggression from patients and families in the ED. Whilst building resilience has been recommended to combat burnout [35], leaders in EDs and health systems have a responsibility to address external causes of burnout.

Meso‐level interventions we identified focussed on ED processes to expedite care, and facilities, environments and staff to improve patient experience while waiting, particularly during periods of overcrowding and long wait times. Simple solutions that could improve patient comfort in the waiting room included better seating and access to food, blankets and television, as well as processes for initiating interventions and pain relief. Nurse‐initiated analgesia and interventions at ED triage have long been promoted for improving care efficiencies and patient flow [37], however, may be restricted by state laws and regulations [38]. Another recommendation for improving efficiency was the use of ticket machines when patients first arrive. Whilst there is little published on the use of electronic ticketing specifically, self‐check‐in kiosks in the ED have been shown to improve triage efficiency [39]. Their use should be considered with regard to time‐sensitive ED presentations, in populations with poor technological proficiency or difficulties reading, and patient preference.

Staffing considerations were prominent in meso‐level recommendations. The need for adequate nurse‐to‐patient staffing ratios in waiting room areas (which are typically limited to the main ED), additional triage nurses during periods of surge and allocated time in workloads for providing person‐centred care were recommended. Insufficient time due to heavy workloads and clinical demand are recognised threats to providing person‐centred care [10], and was a barrier reported by triage nurses in this study. Burnout, emotional fatigue, insufficient experience and lack of organisational support can also threaten the provision of person‐centred care [10]. Possible strategies we identified included rotating staff allocations to triage roles, allocating new or junior nurses with experienced staff for role modelling and mentorship, facilitating staff access to wellbeing services and leadership for person‐centred care. Additional staffing, including waiting room nurses and non‐nursing roles such as concierge, volunteers, Aboriginal Health Workers and security officers, was also recommended and can provide valuable support to both patients and nurses. Specific waiting room nurse roles are a viable option, as they can provide both therapeutic and holistic, person‐centred care to patients in the waiting room [40].

At the macro level, systems and standards to guide ED and nurses deliver person‐centred care were recommended. In Australia, national quality standards for ED services and triage education are used to guide ED and triage structures, processes and practices [41, 42]. These documents mostly focussed on triage systems and requirements, with limited guidance for providing person‐centred care. We identified that guidelines and training should outline requirements and methods for delivering person‐centred care, specifically, such as staffing standards for triage and the waiting room, maximum wait times, recommended waiting room facilities, standards for providing wait time information and consumer involvement in triage policies and design. A lack of stakeholder involvement in ED design processes has been acknowledged internationally, and was proposed by Abdelsamad et al. [43] and McGee [44] to improve patient safety, flow and experience. With continued issues of ED overcrowding and prolonged wait times [3], it is timely to draw attention to the needs and experiences of patients in the waiting room in national guidelines.

### 4.1 Strengths and Limitations

A key strength of the research was the active involvement of and collaboration with consumers throughout the design, conduct and interpretation of results. Patient and nurse representatives provided valuable perspectives and insights for practical application of the research findings [24, 26]. The research was conducted with consumers, for consumers. Participation of both patients and nurses ensured their voices were heard in the research to support a balanced perspective of the phenomena. Collaboration and participation of stakeholders in the research has enabled development of a framework and interventions that can be applied to real‐world settings. The use of a synergistic, fully integrated mixed methods approach allowed comprehensive data collection to ensure findings were authentic, balanced and evidence‐based. Whilst structured, the approach allowed flexibility to adapt research methods to suit contextual changes and challenges over the 5‐year period, including the COVID‐19 pandemic. A limitation was the discontinuation of patient interviews, which may have provided rich data about patients' experiences. However, as well as the single interview, qualitative data were captured in an open‐ended survey question. The research was conducted in the Australian context and some findings may not have wider generalisability. Though patient experience results as well as barriers and facilitators to person‐centred care reflected those published in other countries [6, 10], suggesting that despite local differences, solutions may be applicable broadly.

## 5 Conclusion

We co‐designed a framework for providing person‐centred care in the ED triage and waiting room. Challenges of ED overcrowding and prolonged waits, patients' experiences, as well as the need for support for nurses to provide quality care in these contexts, warrant urgent attention. We identified a range of practical person‐centred interventions that can be implemented at micro, meso and macro levels. Better staffing, waiting room nurse roles, comfortable seating, provision of wait time information and standardised facilities and processes that are designed in collaboration with stakeholders are key recommendations. The simplicity of the framework and variety of interventions allow for broad application beyond the Australian context. Researchers can use the framework to identify interventions in other countries and develop implementation strategies. Future projects should continue O'Cathain and colleagues' process for health intervention development [24] to create, refine, document and evaluate interventions for person‐centred care at ED triage and waiting room.

## Author Contributions

**Carrie Janerka:** conceptualisation, methodology, formal analysis, investigation, data curation, writing – original draft, visualisation, project administration, funding acquisition. **Gavin D. Leslie:** conceptualisation, methodology, validation, formal analysis, writing – review and editing, supervision. **Fenella J. Gill:** conceptualisation, methodology, validation, formal analysis, writing – review and editing, supervision. **PCC ED Triage Group:** methodology, validation, formal analysis, writing – review and editing, visualisation.

## Ethics Statement

The research was approved by the South Metropolitan Health Service Human Research Ethics Committee (RGS0000006844) and reciprocal approval from Curtin University HREC (HRE2024‐0326).

## Consent

All participants were provided an information sheet and gave informed consent (written or implied), including parents or guardians where relevant.

## Conflicts of Interest

The authors declare no conflicts of interest.

## Patient or Public Contribution

Patient representatives with lived experience of ED triage were members of the project's consumer reference group and actively contributed to research design, recruitment strategies, interpretation of findings, framework development and review of the manuscript. Patients and patient representatives participated in the research (survey and focus groups) to share their lived experiences.

## Data Availability

Data generated or analysed during this study are available from the corresponding author upon reasonable request.
